# Transmission Optimization of Social and Physical Sensor Nodes via Collaborative Beamforming in Cyber-Physical-Social Systems

**DOI:** 10.3390/s18124300

**Published:** 2018-12-06

**Authors:** Xuecai Bao, Hao Liang, Longzhe Han

**Affiliations:** 1 Jiangxi Province Key Laboratory of Water Information Cooperative Sensing and Intelligent Processing, Nanchang Institute of Technology, Nanchang 330099, China; 2 Department of Electrical and Computer Engineering, University of Alberta, Edmonton, AB T6G 1H9, Canada; 3 School of Information Engineering, Nanchang Institute of Technology, Nanchang 330099, China; longzhehan@gmail.com

**Keywords:** collaborative beamforming, cyber-physical-social system, social sensor nodes, wireless sensor network

## Abstract

The recently emerging cyber-physical-social system (CPSS) can enable efficient interactions between the social world and cyber-physical system (CPS). The wireless sensor network (WSN) with physical and social sensor nodes plays an important role in CPSS. The integration of the social sensors and physical sensors in CPSS provides an advantage for smart services in different application areas. However, the dynamics of social mobility for social sensors pose new challenges for implementing the coordination of transmission. Furthermore, the integration of social and physical sensors also faces the challenges in term of improving energy efficiency and increasing transmission range. To solve these problems, we integrate the model of social dynamics with collaborative beamforming (CB) technique to formulate the transmission optimization problem as a dynamic game. A novel transmission scheme based on reinforcement learning is proposed to solve the formulated problem. The corresponding implementation of the proposed transmission scheme in CPSS is presented by the design of message exchange processes. The extensive simulation results demonstrate that the proposed transmission scheme presents lower interference to noise ratio (INR) and better signal to noise ratio (SNR) performance in comparison with the existing schemes. The results also indicate that the proposed method has effective adaptation to the dynamic mobility of social sensor nodes in CPSS.

## 1. Introduction

Cyber-physical-social system (CPSS) is attracting increasing attention through the integration of social system and cyber-physical system (CPS). The CPSS as a novel paradigm enables the deep interaction of social space and CPS or Internet of Things (IoT), which brings significant changes for improving the service and management of complex physical systems. Architectures, methods and schemes of CPSS for different application areas have been proposed [1,2,3,4,5,6,7,8,9]. The wireless sensor network (WSN) as the sensing network is one of the most important parts. Importantly, compared with CPS or IoT, the CPSS contains a large number of social sensors, such as smart phones and tablets. The social sensors have a great impact on data collection, transmission, and processing. The novel WSN composed of social and physical sensors undertakes the collection task of sensing information and plays an important role in CPSS. Since the sensor nodes are battery-powered and the battery capacities are limited, improving energy-efficiency of transmission for the novel WSN in CPSS is still one of the most important research topics. In recent years, the emerging collaborative beamforming (CB) technique provides a novel solution for improving energy efficiency of information transmission in traditional WSN. However, due to the characteristic of dynamic social mobility for social sensors, the related studies of CB in traditional WSN composed of physical sensors are not applicable for the CPSS. Our objective was to integrate the social dynamic model with collaborative beamforming technique to solve the problem of coordinated transmission of WSN with social and physical sensors for the CPSS.

In CPS, the traditional WSN only considering fixed physical sensors is widely used to sense or collect the large-scale information due to the characteristics of low cost, lower power and the small devices. The energy efficient schemes for the traditional WSN have been paid great attention in the past few decades. In recent years, the emerging of collaborative beamforming (CB) technique brings a novel solution for improving the energy efficiency of WSNs [10]. The CB technique requires that all fixed sensor nodes in WSN divide into different clusters. All fixed sensor nodes in a cluster transmit collaboratively the sensing information to the intended base station (BS). In process of transmission, the phase synchronization is achieved by the existing synchronization methods. The CB can enhance the transmission gain in intended direction and reduce the interference power at other directions. In other words, these fixed sensor nodes in CB can obtain the beampattern with stable mainlobe and low sidelobe by the cooperative way. Since the sensor nodes are randomly deployed in the given areas, the random distribution of sensor nodes cause that the amplitude of sidelobe in beampattern presents the unpredictability [11,12,13]. It means that the node locations have an important impact for the amplitude of sidelobe [14]. However, the high sidelobe level can lead to the strong interference for the others unintended BSs. Therefore, achieving the required sidelobe level can effectively improve the service performance of CPS.

On the other hand, the existing CB optimization methods for sidelobe control in WSN mainly consider the fixed and static sensor nodes. These methods generally include transmission coefficient optimization [15,16,17,18,19,20] and sensor node selection [21,22,23]. These methods integrate the coefficient optimization into node selection. They first use the intelligence algorithm to optimize the peak sidelobe level and then select an optimal sensor node set based on the regular antenna array. However, these research works have not presented the analysis of the computational complexity and the implementation scheme of information transmission.

As mentioned above, although the existing algorithms exhibit substantial improvement in terms of minimizing the peak sidelobe or sidelobe control in direction of unintended BS, they assume the sensor nodes in traditional WSN are static and the optimization results may cause some sensor nodes to be selected more often such that the network lifetime is reduced. Due to the characteristic of the social dynamic of social sensors for the novel WSN in CPSS, the existing CB optimization methods are not applicable for the novel WSN including mobile social and static physical sensors in CPSS. In this paper, we integrate the social dynamic model into CB to solve the problem. The corresponding transmission optimization method based on reinforcement learning is developed to improve the transmission efficiency of WSN in CPSS. Moreover, the communication mechanism of implementing the transmission scheme and the detailed performance analysis are presented.

The remainder of this paper is organized as follows. The related work is presented in Section 2. Section 3 presents the WSN sensing scenario based on CB in CPSS, including the model of social dynamic mobility and the model of CB transmission. In Section 3, we formulate the CB transmission optimization problem. Section 4 presents a transmission schemes of CB based on reinforcement learning to maximize the SNR performance, while considering the constraint of the interference power at the direction of unintended BS in CPSS. Section 5 evaluates the proposed methods via extensive simulations. Finally, we conclude this work in Section 6.

## 2. Related Work

In recent years, cyber-physical-social system (CPSS) attracts a great amount of attention because it considers the integration of the human activity with CPS or IoT. Many applications based on CPSS have been developed, such as intelligent transportation [2] and smart city [3]. The corresponding architecture, methodology, control, and command schemes are also proposed [1,4,5]. In particular, the sensing network architecture based on WSN as an important part of CPSS plays a significant role for sensing, transmitting and collecting information. Since the WSN architecture composed of social and physical sensor nodes is significantly different from that of traditional WSN, the related research works on traditional WSN are not applicable for CPSS due to the characteristics of dynamic social mobility for social sensors. Therefore, some novel methods are proposed to solve the emerging problems in CPSS. For example, a moving centroid based routing protocol is presented to deal with the incompletely predictable cyber devices in cyber-physical-social distributed systems [6]. In [7], the authors studied a fusion scheme of cellular network and wireless sensor for CPSS. To prolong the lifetime of the IoT, a novel rendezvous data routing scheme based on lower sensors is proposed to achieve the data transmission for scalable CPS networking infrastructure [8]. In addition, a new sensor trajectory planning method is proposed to solve the trajectory planning problem for robotic CPSS [9]. However, all the above-mentioned methods have not considered the improvement of transmission efficiency of the novel WSN in CPSS. In [24], the authors presented a framework and infrastructure of collaborative CPS to improve the management efficiency of the increasing number of devices. The importance of collaboration among components is considered.

On the other hand, the collaborative beamforming technique has been studied extensively in recent years, as it can increase the transmission range and improve the energy efficiency. The related research works on CB transmission in WSN can be divide into three classes, with respect to beampattern analysis, sidelobe level optimization, and synchronization scheme. The analysis of beampattern mainly focuses on the performance of mainlobe, sidelobe, and directivity for different sensor node distributions [10]. The sensor nodes distributions mainly include uniform distribution [11], Gaussian distribution [12], and arbitrary distribution [13]. Based on the analysis of beampattern for different nodes distributions, results are obtained for the performance characteristics of different node distributions and the feasibility of the CB technique in WSN. However, for WSN with small-size nodes, the beampattern performance with random node distribution can hardly meet the transmission requirement in practical applications. To address this problem, the researchers mainly focus on the minimization of peak sidelobe level and the sidelobe control at the unintended receiver directions. The two optimization objectives can be achieved by transmission coefficient optimization and sensor node selection. The two classes of methods can be differential according to the characteristic of transmission coefficient. When the transmission coefficient is continuous, the corresponding methods mainly focus on the optimization of transmission power coefficient. If each sensor node in WSN has only two states, i.e., active on or active off, node selection is generally considered to solve the optimization problem. For transmission coefficient optimization, intelligent algorithms are often used to solve the minimum peak sidelobe or maximum energy efficiency problem for CB in WSN. Due to the non-convex characteristics of this optimization problem, intelligence heuristic algorithms are often employed. For example, the authors in [17,21] utilized particle swarm optimization (PSO) and the firefly algorithm (FA) to optimize the amplitude coefficient of each collaborative node, respectively. Then, a collaborative node set is selected from the candidate nodes based on a circular antenna array. Similarly, the authors in [20] utilized the genetic algorithm (GA) to minimize the peak sidelobe amplitude. Furthermore, several coefficient optimization methods are proposed to extend network lifetime. A multi-objective beampattern optimization problem is formulated in [18], and a metaheuristic method is proposed to calculate the transmission coefficient. This method can ensure low peak sidelobe level and energy consumption, in comparison with the existing heuristic algorithms. In [19], a beampattern optimization method based on non-dominated sorting genetic algorithm II (NSGA II) is proposed to prolong network lifetime for CB in WSN, which effectively reduces the energy consumption and improves the performance of the peak sidelobe. The optimization objectives involve the peak sidelobe and beampattern directivity. Since the transmission coefficient is characterized as a continuous variable, these intelligent algorithms generally have high computational complexity and cause redundant energy consumption. In addition, the corresponding implementation schemes are not introduced in details.

To provide another solution, the situation that the transmitter of each node only has two power levels (i.e., zero and maximum power) is considered. The optimization objective is mainly to minimize the interference power at direction of the other unintended BS by determining the nodes states in terms of active or sleep states. The beampattern optimization problem is typically regarded as a node selection problem. As the problem is a non-convex and combinatory in nature, it is difficult to be solved in polynomial time. Heuristic and combinatory optimization methods are utilized to minimize the sidelobe level. In [22], the authors proposed a random node selection method. This method randomly selects *L* nodes from *N* nodes and evaluates whether the sidelobe level of the *N* nodes meets the given requirement in the direction of unintended BS. Then, it needs to repeat the above process until the requirement is met. However, the computational complexity of this method is very high which may significantly increase the energy consumption of sensor nodes. To reduce the computational complexity, a node selection algorithm based on cross-entropy optimization (CEO) is proposed in [23]. The evaluation results show that the CEO algorithm has better sidelobe performance and lower computational complexity than the method proposed in [22]. However, it only considers static physical sensor nodes and can hardly be extended to mobile networks including the CPSS.

In summary, most of the above-mentioned related works tend to use intelligence algorithms to solve the CB optimization problems for WSN in CPS. These methods assume that the physical sensors in WSN are fixed. However, the social sensors have the property of social dynamic mobility, which significantly affect the performance of CPSS. Therefore, in this work, we focus on the integration of social and physical sensors and propose a corresponding CB optimization method to improve the transmission performance in CPSS.

## 3. System Model and Problem Formulation

This section first presents the sensing scenario of WSN for CPSS. Then, the corresponding transmission model of CB is described.

### 3.1. Sensing Architecture of WSN for CPSS

**Figure 1:**
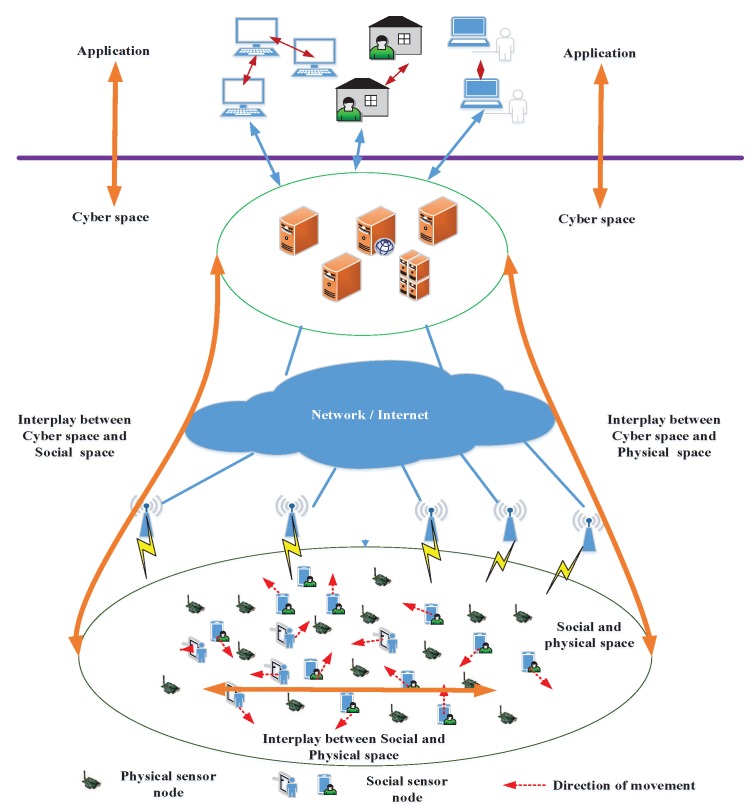
Sensing scenario of WSN in CPSS.

Generally, the CPSS is composed of three parts, i.e., social space, physical space, and cyber space, as shown in Figure 1. The WSN, also called the sensing network, consists of social sensors and physical sensors. The higher abstraction level represents different applications, such as smart home, smart city, intelligent transport and so on. The social and physical sensors in WSN connect to the cyber world through the communication network or Internet. The cyber space interplays with social space and physical space. The social sensor can interact with physical sensors and among each other. The progressive interaction between the two types of sensor nodes can provide the reliable operation, low cost and efficient control for cyber system. They are used to capture the monitoring data and offer the collection of large-scale data. In CPSS, the physical sensors are generally fixed and static. The social sensors are affected by individual affective and physical state [25,26], which is a dynamic process. That is, these social sensors are carried by people and each social sensor moves among different locations based on the social dynamics. It should be noted that the concept of “location” has different definitions in the literature [25,26]. The authors of [25] utilized Voronoi diagram to divide the plane into cells and each Voronoi cell is referred to as a location. In [26], an square cell (block) is a location. In this paper, we consider the definition of location in [25]. In this work, we use the energy efficient CB technique to improve the transmission performance of the social and physical sensing network for CPSS. The corresponding transmission model based on CB is presented in this section.

### 3.2. Model of Social Dynamic Mobility

As we can see in Figure 1, the mobility of social sensors such as mobile phones and tablets is determined by the decisions and behaviors of their owners. Accordingly, their mobility is consistent with the human mobility. In the literature [27,28], several models are proposed to characterize human mobility trajectories. However, these models lack the capability of capturing the features of human dynamic mobility. In [25], the analytical results show that the human movements are not random. Thus, a novel model is proposed to characterize the mobility of humans. The proposed model presents high predictability of human movement. In the following, we present the detailed model of social dynamic mobility.

From the work in [25,26], we know the human trajectories can be characterized by two probability distributions, related to the jump length (Δr) and waiting time (Δt), respectively, given by [25,26]:

(1) $$\begin{array}{cc} P(\Delta r) & ={\left(\Delta r+\Delta r_{0}\right)}^{-1-\alpha }e^{-\Delta r/k_{1}} \end{array}$$

(2) $$\begin{array}{cc} P(\Delta t) & ={\left(\Delta t\right)}^{-1-\beta }e^{-\Delta t/k_{2}} \end{array}$$

where the jump length Δr is the distance from current location to the next location. The waiting time Δt denotes the spending time of individual at the same location. α and β are the parameters controlling the jump distance and wait time, respectively, while $k_{1}$ and $k_{2}$ are the cutoff values of jump distance and wait time, respectively. The value of $\Delta r_{0}$ specifies the minimum jump distance.

According to the above distributions and the analysis in [25], the individual will change the current location after a waiting time Δt. The individual mobility process is described by two generic mechanisms, i.e., exploration and preferential return. For exploration, each social sensor node intends to move to a new location with probability $P_{new}$

(3) $$P_{new}=\rho S^{-\gamma }.$$

In Equation (3), the *S* denotes the number of visited locations, while ρ,γ refer to the parameters of exploring a new location and returning to a visited location, respectively. The social sensor node visits the new location based on the probability $P_{new}$. After a waiting time Δt, the social sensors return to the previously visited location based on the following probability $P_{ret}$

(4) $$P_{ret}=1-\rho S^{-\gamma }.$$

The analytical results for the model in [25] show that, over time, the number of visited locations *S* should follow the law:

(5) $$S\left(t\right)∼t^{\mu }.$$

where μ=β/(1+γ), and γ refers to the parameter of returning to a visited location, γ>0. β denotes the parameter of controlling wait time, 0<β<1. We know that μ<1, which means that the S(t) has a decreasing tendency of visiting unvisited locations for the individual. According to the above definition of location in Section 3.1, the number of locations in a city or urban area is limited. When the simulation time *t* tends to be infinity, the number of visited location will be constant. Then, based on the initial time moment, we can determine the number of visited locations *S*. According to the analysis in [25], we can count the frequency of visiting location *i* and calculate the corresponding transition probability between any two visit locations *j* and *k*, i.e., $P_{i}\left(s_{j}|s_{k}\right)=F_{j,k}^{i},i\in \left\{1,\cdots ,O\right\},j,k\in \left\{1,2,\cdots ,S\right\},j\neq k$.

(6) $$P_{i}\left(s_{j}|s_{k}\right)=\left[\begin{array}{cccc} 0 & F_{\left(1,2\right)}^{i} & \cdots & F_{\left(1,S\right)}^{i}\\ F_{\left(1,2\right)}^{i} & 0 & \cdots & F_{\left(1,S\right)}^{i}\\ \vdots & \vdots & \ddots & \vdots \\ F_{\left(S,1\right)}^{i} & F_{\left(S,2\right)}^{i} & \cdots & 0 \end{array}\right]$$

From the work in [25], we know that the model can effectively capture the basic features of human social mobility. Therefore, we use this model to characterize the mobility of social sensors. In this work, since the number of the visited locations for human in a area is limited, we utilize the above model to establish a fixed number of visited locations, and then each social sensor returns to the previous visited location based on the above transition probability.

### 3.3. Transmission Model of CB

**Figure 2:**
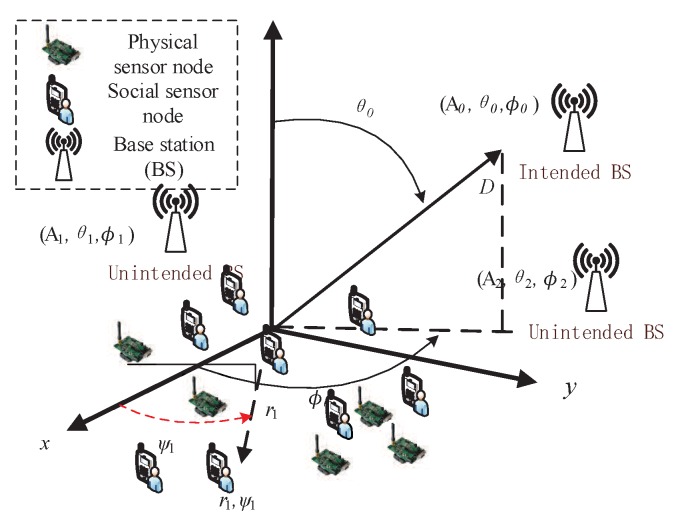
Social and physical sensing architecture based on CB for CPSS.

We consider a CPSS with a social or physical sensing WSN with *M* nodes, in which there are *O* social sensor nodes and *F* physical sensor nodes randomly deployed on a plane, as shown in Figure 2. We assume *D* base stations (BSs) are located in the surrounding area of the WSN. We use $R=\{R_{0},R_{1},\cdots ,R_{D}\}$ and $Z=\{z_{1},z_{2},\cdots ,z_{M}\}$ to denote the BSs and the sensor nodes, respectively. The nodes $\left\{z_{1},z_{2},\cdots ,z_{O}\right\}$ are referred to as the social sensor nodes. The other nodes denote the physical sensor nodes. The transmission range of each sensor node cannot fully cover the BS due to the limited transmission power. As a result, the sensor nodes need to use CB technique to transmit the monitoring information to the intended BS. Moreover, the polar coordinates $\left(r_{k},\psi_{k}\right)$ and $\left\{\left(A_{0},\theta_{0},\varphi_{0}\right),\left(A_{1},\theta_{1},\varphi_{1}\right),\cdots ,\left(A_{D},\theta_{D},\varphi_{D}\right)\right\}$ are used to denote the location of the $k_{th}$ collaborative node and BS, respectively. We use $d_{k}\left(\varphi ,\theta \right)$ to denote the Euclidean distance between the *k*th node and location (A,θ,φ). The elevation angle θ lies in the range from 0 to π and the corresponding azimuth angle is φ is from -π/2 to π/2. According to the literature [11], we first present the value of $d_{k}\left(\varphi ,\theta \right)$ as follows

(7) $$d_{k}\left(\varphi ,\theta \right)=\sqrt{A^{2}+r_{k}^{2}-2r_{k}A\sin\theta \cos\left(\varphi -\psi_{k}\right)}$$

When the locations of *M* collaborative nodes are obtained, the corresponding array factor is given by

(8) $$AF\left(\varphi ,\theta ,\mathrm{cP}\right)=\sum_{k=1}^{O}c_{k}\sqrt{P_{k}}e^{j\Psi_{k}}e^{j\frac{2\pi }{\lambda }d_{k}\left(\varphi ,\theta \right)}+\sum_{i=1}^{F}c_{i}\sqrt{P_{i}}e^{j\Psi_{i}}e^{j\frac{2\pi }{\lambda }d_{i}\left(\varphi ,\theta \right)}$$

where *O* denotes the number of social collaborative nodes, and *F* is the number of physical sensor nodes. In addition, $P_{k}$ refers to the transmitting power of the *k*th node $z_{k}$, while λ is the wavelength of signal carrier. Let the initial phase of the *k*th node be:

(9) $$\Psi_{k}=-\frac{2\pi }{\lambda }d_{k}\left(\varphi ,\theta \right)$$

Then, the corresponding array factor AF at the *l*th BS can be rewritten as [11]:

(10) $$AF\left(\varphi_{l},\theta ,\mathrm{cP}\right)=\sum_{k=1}^{O}c_{k}\sqrt{P_{k}}e^{j\Psi_{k}}e^{j\frac{2\pi }{\lambda }d_{k}\left(\varphi_{l},\theta \right)}+\sum_{i=1}^{F}c_{k}\sqrt{P_{i}}e^{j\Psi_{i}}e^{j\frac{2\pi }{\lambda }d_{i}\left(\varphi_{l},\theta \right)}$$

Similar to Mudumbai [11], we focus on the radiation pattern in the far-field region. Thus, we also assume that the far-field condition holds, i.e., the distance between the origin of coordinates and the *l*th base station is far greater than the $r_{k}$, $A_{l}\gg r_{k}$. Then, $d_{k}\left(\varphi_{l},\theta \right)$ can be approximated as

(11) $$d_{k}\left(\varphi_{l},\theta \right)=\sqrt{A^{2}+r_{k}^{2}-2r_{k}A\sin\theta \cos\left(\varphi -\psi_{k}\right)}\approx A_{l}-r_{k}\cos\left(\varphi_{l}-\psi_{k}\right)$$

Without loss of generality, θ is set to π/2, which means the BS is in the same plane with the sensor nodes. When the value of the initial phase in Equation (9) is met by the phase synchronization technique, the amplitude of AF at the direction of the intended $BS_{0}$ can be rewritten as

(12) $$AF\left(\varphi_{0},\mathrm{cP}\right)=\sum_{k=1}^{O}c_{k}\sqrt{P_{k}}+\sum_{i=1}^{F}c_{i}\sqrt{P_{i}}$$

Next, we present the description of the received signal at intended BS and unintended BS. We assume that all collaborative nodes receive the sending data symbol $m_{i}$ from source node, where $m_{i}\in C$ and satisfies $E\{m_{i}\}=0$, $|m_{i}{|}^{2}=1$, and $E\{m_{i}m_{i^{\prime }}\}=0$ for $i\neq i^{\prime }$, where E{·} denotes the expectation operation. Furthermore, the channel coefficient $h_{kl}$ is assumed to follow a lognormal distribution [23], i.e., $h_{kl}∼exp\left\{M\left(0,\sigma^{2}\right)\right\}$, where $\sigma^{2}$ is the variance of the corresponding distribution. Then, the formula of the received signal at BS can be obtained as

(13) $$x\left(\varphi_{l},\mathrm{cP}\right)=\sum_{k=1}^{O}m_{k}c_{k}\sqrt{P_{k}}h_{kl}e^{j\Psi_{k}}e^{j\frac{2\pi }{\lambda }\left(A_{l}-r_{k}\cos\left(\varphi_{l}-\psi_{k}\right)\right)}+\sum_{i=1}^{F}m_{i}c_{i}\sqrt{P_{i}}h_{il}e^{j\Psi_{i}}e^{j\frac{2\pi }{\lambda }\left(A_{l}-r_{i}\cos\left(\varphi_{l}-\psi_{i}\right)\right)}+\omega$$

where $\omega ∼CN(0,\sigma_{\omega }^{2})$ refers to the additive white Gaussian noise. According to Equation (12), the received signal at the intended ${\mathrm{BS}}_{0}$ can be expressed as

(14) $$s\left(\varphi_{0},\mathrm{cP}\right)=\sum_{k=1}^{O}m_{k}c_{k}\sqrt{P_{k}}h_{k0}+\sum_{i=1}^{F}m_{i}c_{i}\sqrt{P_{i}}h_{i0}+\omega .$$

For the unintended BSs, the received signal can be written as

(15) $$s\left(\varphi_{l^{\prime }},\mathrm{cP}\right)=\sum_{k=1}^{O}m_{k}c_{k}\sqrt{P_{k}}h_{kl^{\prime }}e^{j\Psi_{k}}e^{j\frac{2\pi }{\lambda }\left(A_{l^{\prime }}-r_{k}\cos\left(\varphi_{l^{\prime }}-\psi_{k}\right)\right)}+\sum_{i=1}^{F}m_{i}c_{i}\sqrt{P_{i}}h_{il^{\prime }}e^{j\Psi_{i}}e^{j\frac{2\pi }{\lambda }\left(A_{l^{\prime }}-r_{i}\cos\left(\varphi_{l^{\prime }}-\psi_{i}\right)\right)}+\omega$$

where $l^{\prime }\neq 0$. Correspondingly, the received interference power at the unintended BSs is given by

(16) $$I\left(\varphi_{l^{\prime }},\mathrm{cP}\right)={\left|\sum_{k=1}^{O}m_{k}c_{k}\sqrt{P_{k}}h_{kl^{\prime }}e^{j\Psi_{k}}e^{j\frac{2\pi }{\lambda }\left(A_{l^{\prime }}-r_{k}\cos\left(\varphi_{l^{\prime }}-\psi_{k}\right)\right)}+\sum_{i=1}^{F}m_{i}c_{i}\sqrt{P_{i}}h_{il^{\prime }}e^{j\Psi_{i}}e^{j\frac{2\pi }{\lambda }\left(A_{l^{\prime }}-r_{i}\cos\left(\varphi_{l^{\prime }}-\psi_{i}\right)\right)}\right|}^{2}$$

According to Equation (15), the signal-to-noise ratio (SNR) at the intended ${\mathrm{BS}}_{0}$ can be calculated as

(17) $$S\left(\varphi_{0},\mathrm{cP}\right)=\frac{|s\left(\varphi_{0},\mathrm{cP}\right){|}^{2}}{\sigma_{\omega }^{2}}.$$

The interference-to-noise ratio (INR) at all unintended BSs can be written as

(18) $$\mathrm{INR}\left(\varphi_{l},\mathrm{cP}\right)=\frac{1}{D}\sum_{l=1}^{D}\frac{|I\left(\varphi_{l},\mathrm{cP}\right){|}^{2}}{\sigma_{\omega }^{2}}.$$

### 3.4. Problem Formulation

In this section, we formulate the transmission optimization problem. For a WSN using CB technique in CPSS, the following features should be considered. Firstly, since social sensor nodes have dynamic mobility, the CB transmission in WSN needs to consider the integration of the fixed physical sensors and the mobile social sensor nodes. Secondly, the location of each node determines the CB performance. Thus, the proposed method is required to capture the dynamic mobility for social nodes and reduce its impact on the beampattern performance of CB in WSN. In addition, to increase data transmission rate, the SNR performance at the intended BS needs to be considered, and the INR at unintended BS is required to meet the specific requirement. From Equation (9), if the transmission power of each node only has two levels, i.e., zero and $P_{max}$, then the number of the selected collaborative nodes determines the value of SNR. Therefore, the objective of our transmission optimization problem is to maximize the SNR at intended BS while considering the dynamic mobility of social sensor nodes, given the requirement that the INR at unintended BS should be greater than a specific value $INR_{0}$. The optimization problem can be formulated as follows:

(19)maxc∑t=1TS(φ0,cP)(20)subjecttoINR(φl,cP)<INR0(21)Pi(sj|sk)=Fjki,i∈1,⋯,O(22)ci=0or1,i=1,2,...,M

where **c**=$\left\{c_{1},c_{2},\cdots ,c_{M}\right\}$ (with $c_{i}\in \left\{0,1\right\}$ the vector of node selection), $c_{i}=1$ denotes that the *i*th node is selected into the candidate collaborative node set, and $F_{jk}^{i}$ represents the probability from the location *k* return to location *j* for social sensor node $z_{i}$. In Equation  (19), we assume the time period is divided into *T* time slots. Thus, the *T* in Equation (19) denotes the number of time slots in the time period. From above objective function, we can see that this is a non-linear and non-convex optimization problem. As a result, it is difficult to derive an optimal algorithm to solve this problem as the computational complexity grows exponentially with the number of nodes and BSs. Furthermore, the location of social sensor nodes is changing based on the probability of social dynamic mobility $F_{j,k}^{i}$, which is stochastic in nature. At the same time, the characteristics of social dynamic mobility for social sensor nodes have great impacts on the INR. To address these issues, we use a stochastic game approach in this paper to maximize the SNR performance under the INR constraint in the directions of unintended BSs ($INR_{0}$). In addition, considering the practical implementation WSN, it is necessary to develop an effective learning algorithm to tackle the social dynamic mobility.

## 4. The Propose Transmission Optimization Algorithm

In this section, we solve the above-mentioned transmission optimization problem based on a dynamic learning algorithm. Then, the corresponding implementation scheme is presented.

### 4.1. Dynamic Learning Algorithm

A game theoretical approached is used to solve this problem. Each sensor nodes in this system acts as a game player to interact with other sensors. In a dynamic environment, the reactive action of the game can obtain better performance than a deterministic method. For the problem under investigation, the location of each social sensor node follows a dynamic process. Hence, we use a dynamic learning algorithm to solve this problem. In this work, we first present the game as $\Gamma =<M,{\left\{A_{i}\right\}}_{i\in M},X,{\left\{\mu_{i}\right\}}_{i\in M}>$, where *M* denotes the number of players, X represents the state of dynamic environment, and $\mu_{i},i=1,\cdots ,M$ is the utility function of the player. In this work, the action set of sensor node includes the two transmission power levels $A_{i}=\left\{0,P_{max}\right\}$, which refer to the sleep and active states of each sensor node, respectively. For the utility function $\mu_{i}$, we first analyze the optimization objective and the constraints. In particular, the objective is to maximize the SNR at the intended BS, which is proportional to the transmission rate. The action of each sensor node, i.e., $c_{i}=1$ or $c_{i}=0$, determines the value of the SNR. When $c_{i}=1$, the transmission power is $P_{max}$, otherwise, it is 0. We consider the INR for the unintended BSs as a penalty term of the objective function. Then, the corresponding utility function $\mu_{i}$ can be derived as follows:

(23) $$\mu_{i}=S\left(\varphi_{0},\mathrm{cP}\right)-\kappa \left(\mathrm{INR}\left(\varphi_{l},\mathrm{cP}\right)-INR_{0}\right).$$

Here, since all *M* sensor nodes use the collaborative beamforming technique to achieve cooperatively the same optimization objective, the utility function of each node is the same, i.e., $\mu_{1}=\mu_{2}=\cdots =\mu_{M}$.

For the game theoretical approach, reinforcement learning methods are generally used to solve the problem. Although the Markov decision process (MDP) can also be used to solve the problem, the accurate state information is required, which is difficult to obtain in practical applications. In addition, the implementation of MDP is centralized. For the CPSS, the state of each social sensor node is determined by human activity. Thus, the accurate prior knowledge is hardly obtained in dynamic environment. In addition, the centralized optimization is hard to be implemented due to the difficulty of collecting the global state information of all sensor nodes. For the WSN with a distributed architecture in CPSS, a distributed scheme needs to be considered. Therefore, we first present a distributed reinforcement learning algorithm based on regret matching to improve the transmission performance of the WSN with social and physical sensor nodes in CPSS. The regret matching scheme focuses on the regret related to the actions of each player. For any time, each player (node) calculates the regret value by adjusting its strategy. The specific definition is given by

(24) $$R_{i}^{a_{i}}\left(t\right)=\frac{1}{t}\sum_{\tau =1}^{t}\left[\mu_{i}\left(a_{i},a_{-i}\left(\tau \right)\right)-\mu_{i}\left(a\left(\tau \right)\right)\right]$$

where $\mu_{i}\left(a\left(\tau \right)\right)$ denotes the received utility for each player at time τ, and $\mu (a_{i},a_{-i}\left(\tau \right))$ is the utility when the player switch to action $a_{i}$ while other players retaining theirs unchanged. Then, each player updates its strategy $p_{i}\left(t\right)$ as follows:

(25) $$p_{i}^{a_{i}}\left(t\right)=\frac{{\left[R_{i}^{a_{i}}\left(t\right)\right]}_{+}}{\sum_{a_{i}^{\prime }\in A_{i}}{\left[R_{i}^{a_{i}^{\prime }}\left(t\right)\right]}_{+}}$$

where ${\left[R_{i}^{a_{i}}\left(t\right)\right]}_{+}=\max\left\{R_{i}^{a_{i}}\left(t\right),0\right\}$.

The proposed algorithm is presented in Algorithm 1, where the regret matching method is used to maximize the utility in Equation (23). In the initialization stage, each social or physical sensor node initializes the location of node, state, and probability of action selection. Then, each sensor node selects the specific action (corresponding to the transmission power) according to the initial probability. After that, the learning function in Equation (24) is used to update the regret value based on the utility function. The probability of action selection is recalculated based on Equation (25). Then, the location of social sensor nodes is changed according to the model of social dynamic mobility in Section 3. Finally, when the number of iteration meets the stopping condition, the result is returned and the algorithm stops. Otherwise, the above procedure is repeated.

**Algorithm 1** The proposed algorithm based on regret matching.
**Input:** The number of sensor nodes *M*; The coordinates of nodes and BSs $\{r_{k},\varphi_{k}\},k=1,2,\cdots ,M$; $R_{l},l=0,1,\cdots ,D$; The number of iteration *T*
**Output:** The probability of action selection $P_{a_{i}}^{i}=1/2,i=1,2,\cdots ,M,a_{i}\in A_{i}$
 1:   Initialization: The regret value of social sensor nodes $R_{i}^{a_{i}}\left(t,n\right)=0$, i∈{1,2,⋯,O},n={1,2,⋯,S}, where *n* is the location label of social sensor node; The regret value of physical sensor nodes $R_{j}^{a_{j}}\left(t\right)=0$, j∈{O+1,O+2,⋯,M}; The probability of action selection $P_{a_{i}}^{i}=1/2$, i=1,2,⋯,M,$a_{i}\in A_{i}$; t=1 2:Calculate the transition probability $F_{jk}^{i},i\in \left\{1,\cdots ,O\right\}$ based on Equations (3)–(5) of the model of dynamic mobility in Section 3.2 3:Repeat 4:   For each sensor node $z_{i}$ do 5:      Select a action $a_{i}$ for $A_{i}$ based on the $P_{a_{i}}^{i}\left(t\right)$ 6:   EndFor 7:   Calculate the utility based on the Equation (23) 8:   For each sensor node $z_{i}$ do 9:   If $z_{i}$ is social node, i∈{1,2,⋯,O}10:      Calculate the corresponding regret value $R_{i}^{a_{i}}\left(t,n\right)$ of different action at location *n* according to Equation (23)11:   else12:      Calculate the corresponding regret value $R_{i}^{a_{i}}\left(t\right)$ of different action using Equation (24)13:   EndIf14:   Update the probability of action selection $P_{a_{i}}^{i}\left(t\right)$ using Equation (25).15:   EndFor16:   Update the location information of each social node based on the transition probability $F_{jk}^{i},i\in \left\{1,\cdots ,O\right\}$ of social dynamic mobility17:   t=t+118:Until t>=T19:**return** the probability of action selection $P_{a_{i}}^{i}$i∈{1,2,⋯,M}


In Algorithm 1, some initial variables are initialized on Line 1. The calculation of transition probability is presented on Line 2. On Lines 3–6, each node selects a action from action set based on the probability of action selection, i.e., selecting a transmission power level (zero or $P_{max}$). Then, the corresponding utility is calculated based on Equation (23) on Line 7. On Lines 8-013, the regret value $R_{i}^{a_{i}}\left(t,n\right)$ or $R_{i}^{a_{i}}\left(t\right)$ for social or physical sensor node is calculated based on Equation (24). Based on the regret value, the probability of action selection of each node is recalculated according to Equation (25). Next, the location of social sensor node is updated based on the transition probability of social dynamic mobility. Note that the location for physical sensor node is static. Thus, the location is unchanged. After this, it returns to Line 3 to repeat the calculation procedure. On Lines 18–19, when t>T, the algorithm stops and returns the probability of action selection of each node.

### 4.2. The Implementation Scheme

To implement the proposed CB transmission scheme of sensor nodes in CPSS, we present the message exchange process for communications. Then, the proposed algorithm can be implemented in a decentralized way. Let $C_{Z}$ be collaborative node set with *O* social sensor nodes and (M-O) physical nodes; our objective is to select a collaborative node set from *M* candidate nodes including social and physical sensor nodes. The detail implementation procedure is given as follows:

**Step 1: Initialization.** Source node *s* sends the Initialization Messages to each node $z_{m}$ (m=1,2,...,M) in the deployment area. Each node initializes its regret value by $R_{i}^{a_{i}}\left(t,l\right)=0$ or $R_{i}^{a_{i}}\left(t\right)=0$ with i∈{1,2,⋯,O} and l={1,2,⋯,S}, as well as the probability of action selection $P_{a_{i}}^{i}=1/2$. The iteration index is set to t=1.

**Step 2: Collect Node Information.** Source node *s* sends an Information Collection Message to each node $z_{m}$ (m=1,2,...,M) in the deployment area. When a node receives the Information Collection Message, its sends the State Information Message including its node location, node ID, and current strategy as a response to source node. The source node saves the state information of all candidate nodes and broadcasts the information to each sensor node and then carries out the next step.

**Step 3: Establish Collaborative Node Set.** Source node sends Node Selection Messages to all social and physical sensor nodes in transmission range of source node. When the sensor node $z_{i}$ can receive the message, it selects an action $a_{i}$ according to the probability of action selection and responds by an Action Selection Message. This step repeats until all neighbouring sensor nodes of the source node have responded. Then, the selected nodes use CB to transmit the State and Strategy Information to the intended BS.

**Step 4: Calculate the Utility.** The intended BS calculates the utility according Equation (23). Then, the intended BS returns the result through an Utility Information Message to each collaborative node.

**Step 5: Calculate Regret Value and Update the Probability of Action Selection.** Each node calculates the regret value based on the received utility using Equation (24). Then, the probability of action selection are updated using Equation (25).

**Step 6: Update the Social Sensor Node Location.** The social sensor nodes update the current location information based on the social dynamic mobility.

**Step 7: Repeat Iteration.** If the number of iterations is less than the maximum number, then t=t+1 and return to Step 2. Otherwise, the learning process stops and go to the next step.

**Step 8: Data Transmission.** Source node *s* broadcasts the Sensing Information Message to all collaborative nodes with $c_{i}=1$. Then, each candidate node responds by a confirmation Message. Then, the phase synchronization and CB technique are used to send the data symbol to the intended BS. When the intended BS successfully receives the signal and the interference power at the unintended BS is lower than the required value, each collaborative node will implement the transmission of data symbol based on the current strategy.

### 4.3. The Complexity of the Proposed Algorithm

Here, we analyze the complexity of the proposed algorithm. According to the description of implementation for the proposed algorithm, each sensor node needs to execute the following operations. First, the transition probability is required to be calculated. In this procedure, we consider the complexity of the calculation is *U*. Moreover, the main calculation in Algorithm has the calculation of utility in Equation (23) and regret value in Equation (24). The corresponding complexity is denoted by $C_{u}$ and $C_{r}$. We know the iteration number is *T*. Thus, the total computational complexity is $U+T(C_{u}+C_{r})$. In terms of energy consumption, we assume the consumed energy of the computation is $E_{c}$. In procedure of implementation, the messages mainly have Information and State Collection Messages, Node and Action Selection Messages, State and Strategy Information Messages, Utility Information Message, Sensing Information Message, and Confirmation Message. We assume the energy consumptions of these messages are denoted by $E_{IS}$, $E_{NA}$, $E_{SS}$, $E_{UI}$, $E_{SI}$, and $E_{CM}$. The total energy consumption is $E_{c}+E_{IS}+E_{NA}+E_{SS}+E_{UI}+E_{SI}+E_{CM}$. We know from that the CB itself is a technique of improving energy efficiency and the consumed energy of each node can be reduced by an order of $\frac{1}{M}$ for the consumed energy without using CB. From the implementation scheme, the  State and Strategy Information Messages and Sensing Information Message are transmitted to sink node by CB. Therefore, the proposed algorithm can reduce the energy consumption of $\frac{M\;-\;1}{M}\left(E_{SS}+E_{SI}\right)$.

## 5. Performance Evaluation

In this section, we present the performance evaluation of the proposed algorithm for CB in WSN. We know from the work in [10] that the real experimentation of collaborative beamforming in WSN needs the support of carrier, phase, and time synchronization technique. Although various phase synchronization approaches of CB are presented in the literature [10], the synchronization is still under study. Therefore, we assume the synchronization can be performed and use the simulation experiment to evaluate the CB transmission performance of the proposed algorithm in CPSS. We first consider a WSN with 5 social sensor nodes and 25 physical sensor nodes, which are randomly deployed in a circular area with R=2λ radius. We assume that each social sensor node has S=8 locations in this area. The movement is based on the model of the social dynamic mobility. The location of each physical sensor nodes remains unchanged in the whole process. We first consider the INR threshold $INR_{0}=4$ dB. The transmit power of each node has two levels, i.e., zero and $P_{max}=\delta_{\omega }^{2}\xi /N$, where ξ is normalized by $\delta_{\omega }^{2}$. The value of ξ is the same as that in [11,23], which is set as 20 dB. The channel coefficient follows a distribution with a zero-mean and $\sigma^{2}=0.2$. Moreover, the direction of the intended BS is $\varphi_{0}=0^{\circ }$, and other three unintended BSs are located at $\varphi_{1}=-130^{\circ }$, $\varphi_{2}=-50^{\circ }$, and $\varphi_{3}=120^{\circ }$, respectively.

**Figure 3:**
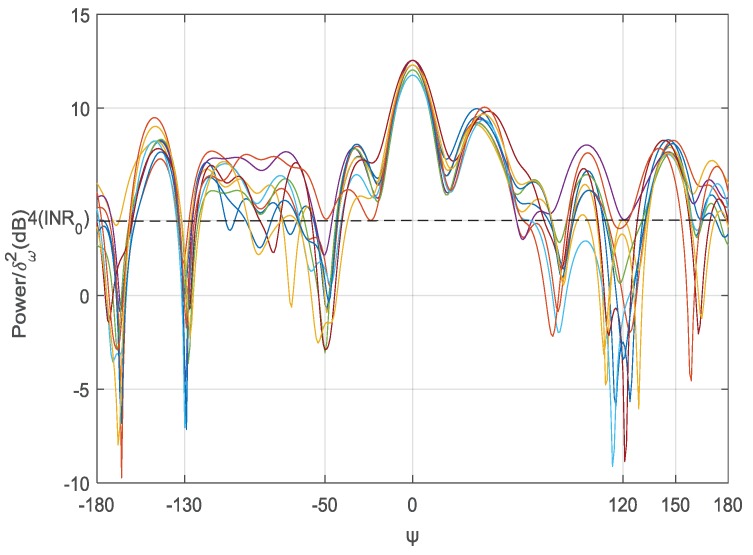
Beam patterns for 10 time slots. The direction of intended BS is $\varphi_{0}=0^{\circ }$, and the other three directions for unintended BSs are $\varphi_{1}=-130^{\circ },\varphi_{2}=-50^{\circ }$, and $\varphi_{3}=120^{\circ }$, respectively.

We first analyze the beam pattern performance. Based on the above setting, we present the beam pattern performance for 10 time slots in Figure 3. As we can see, the interference powers for all three unintended BSs are less than the required $INR_{0}=4$ dB. The result shows the proposed algorithm can adapt to the location change of social sensor nodes.

**Figure 4:**
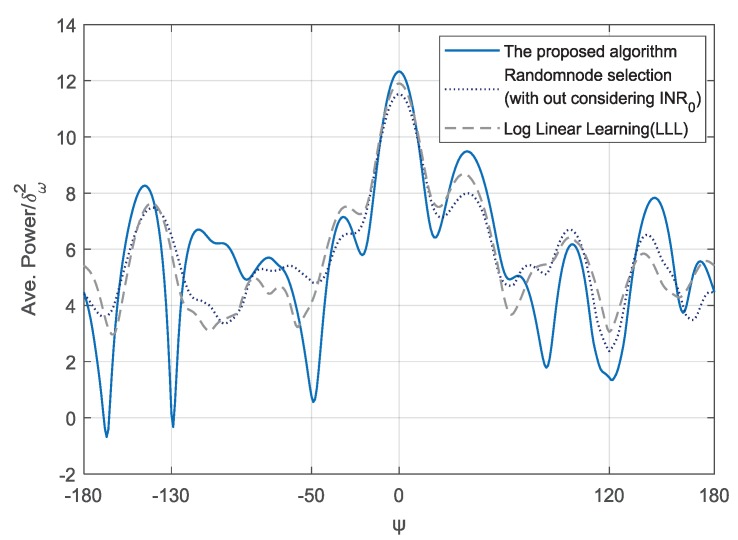
Average beam patterns of 10 time slots for different methods. The direction of intended BS is $\varphi_{0}=0^{\circ }$, and the other three directions for unintended BSs are $\varphi_{1}=-130^{\circ },\varphi_{2}=-50^{\circ }$, and $\varphi_{3}=120^{\circ }$, respectively.

To further analyze the performance, we compare the proposed algorithm with log linear learning (LLL) method [29] and the random node selection algorithm [22]. The LLL is often used to optimize the game theoretical problem [30,31]. The method focuses on the updating of the action selection probability based on the received utility. The random node selection is to choose *L* nodes to meet the requirement of transmission gain in the direction of intended BS or low interference power for the directions of the unintended BSs. The comparison result of average beampattern performance is shown in Figure 4. It can be observed that, when the $INR_{0}$ is set as 4, the result indicates that the INR at the three unintended BSs of the proposed algorithm is much lower than that of the log-linear learning method and the random node selection algorithm. In addition, the SNR of the mainlobe of our proposed algorithm at the direction of the intended BS is much higher than that of the other algorithms. This is because the proposed algorithm considers the utility of all different position for all previous time slots based on regret matching. For the LLL method, the utilities of different actions are calculated and then the action selection probability is updated based on the current utility. Since the location of social sensor node is dynamic, the LLL method can hardly capture such location change.

**Figure 5:**
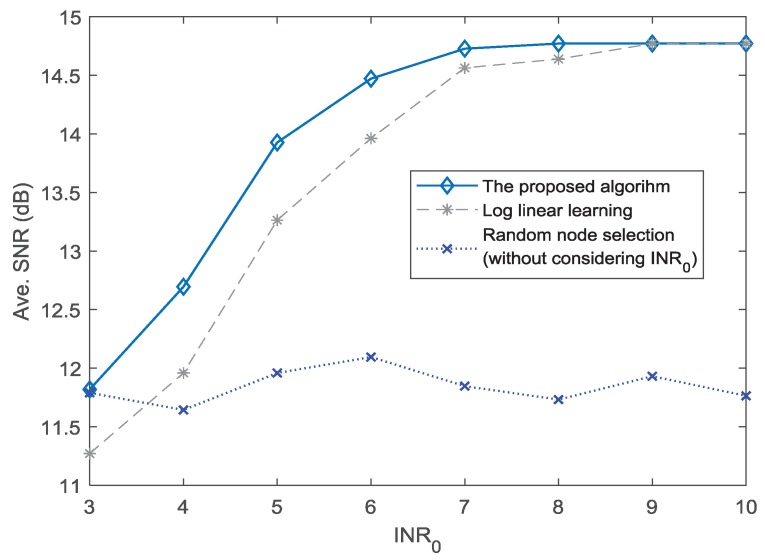
Average SNR performance for different $INR_{0}$.

Figure 5 shows the average SNR performance for different thresholds of $INR_{0}$. It can be seen that the proposed algorithm has higher SNR performance than the other two algorithms. When the $INR_{0}$ is set to 8, the proposed algorithm obtains the maximum SNR amplitude. The LLL method achieves the best performance only when $INR_{0}=9$. In addition, when the $INR_{0}$ is in the range 3–9, the proposed algorithm has a better SNR than the LLL method. Because the method of random node selection can hardly meet the requirement of $INR_{0}$, the SNR performance for the situation without considering $INR_{0}$ falls in the area between 11.5 dB and 12 dB. It can be seen in Figure 5 that the proposed algorithm can effectively improve the SNR performance under the given different $INR_{0}$.

**Figure 6:**
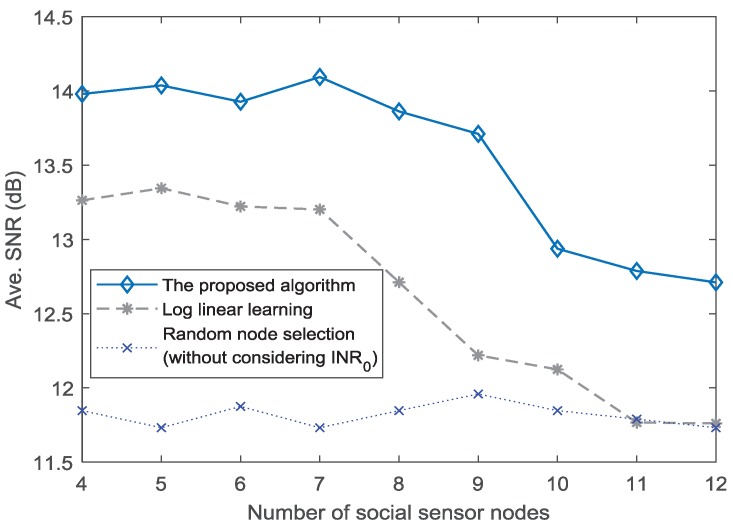
SNR performance for different numbers of social sensor nodes ($INR_{0}=5$ dB).

**Figure 7:**
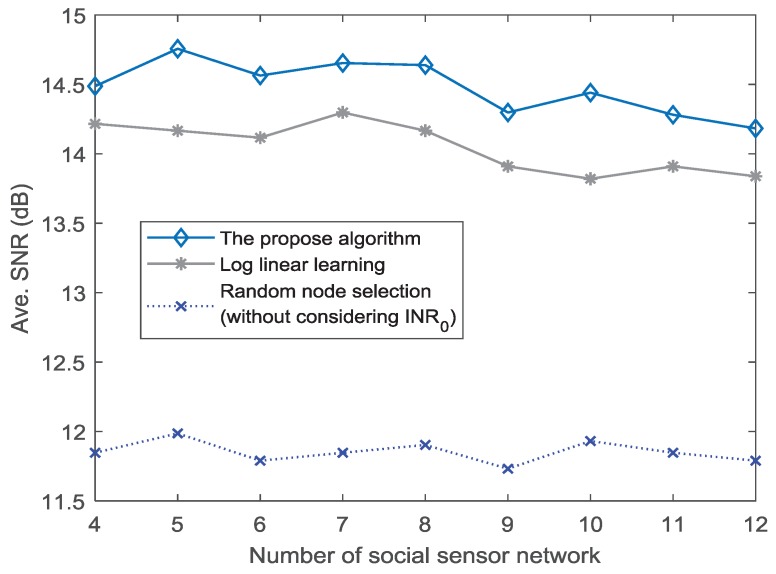
SNR performance for different numbers of social sensor nodes ($INR_{0}=7$ dB).

The above analysis mainly focuses on a fixed number of social and physical sensor nodes. To further analyze the performance with respect to different number of social sensor nodes, we consider 4–12 social sensor nodes. Then, the SNR performance under $INR_{0}=5$ dB is presented in Figure 6. The SNR performance of the proposed algorithm and LLL method decreases with the number of social sensor nodes. Specifically, when the number of social sensor nodes is greater than 9, the SNR amplitude reduces significantly for the proposed algorithm. Similarly, this situation also happens in LLL method. It shows that, although some social sensor nodes integrated into physical sensor nodes have positive role for the CB transmission in CPSS, excessive social sensor nodes have a negative impact on the transmission of CB. The reason is because the movements of excessive social sensor nodes can hardly meet the condition of $INR_{0}$. When the $INR_{0}$ is set as 7 dB, the impact on the transmission of CB is weakened. As shown in Figure 7, the SNR performance reduces slowly with the number of social sensor nodes. It is evident in Figure 6 and Figure 7 that the number of social sensor nodes has an impact on the CB transmission. However, as the value of $INR_{0}$ increases, the impact is weakened gradually.

**Figure 8:**
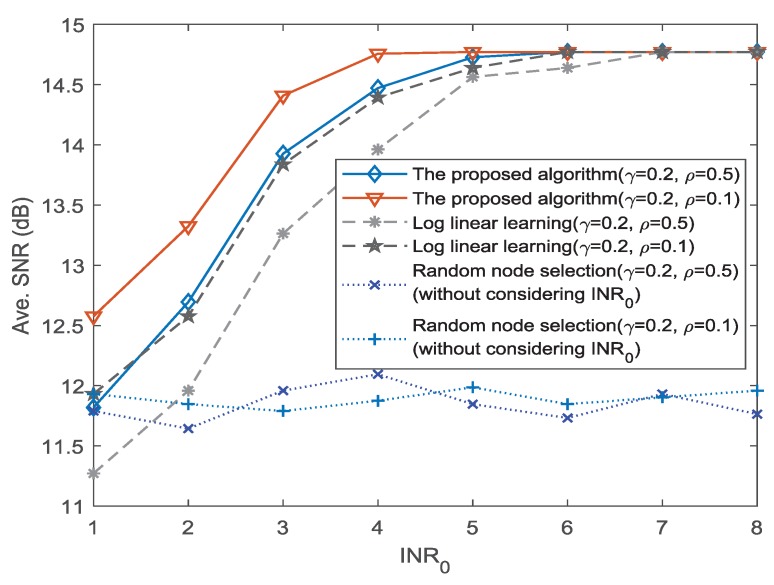
SNR performance for different mobility parameters.

Next, we analyze the influence of different values of γ and ρ for the dynamic mobility model. According to the model of dynamic mobility in [25], we consider two groups of model parameters: (a) γ=0.2 and ρ=0.5; and (b) γ=0.2 and ρ=0.1. The values of γ and ρ can determine the probability of preferential return. Based on the two groups of parameters, we can obtain different transition probabilities of social sensor nodes. The corresponding SNR performance for different mobility parameters is shown in Figure 8. It can be seen that the SNR performance when ρ=0.1 is better than that of ρ=0.5, for both the proposed algorithm and the LLL method. As the increase of the threshold $INR_{0}$, the difference between the two groups of parameters reduces gradually. This is due to the following reasons. First, ρ=0.1 means each social sensor node has greater probability to return to the previously visited nodes than that of ρ=0.5. It reduces the difficulty of capturing the dynamic mobility for the proposed learning method. Second, as the value of the threshold $INR_{0}$ increases, the constraint is weakened. The proposed algorithm can adapt to the change such that the difference of the SNR performance is reduced.

## 6. Conclusions

In the CPSS, the WSN with both social and physical sensor nodes plays an important role in terms of data monitoring and sensing. Due to the characteristics of dynamic mobility for the social sensor nodes, the transmission optimization methods of traditional WSN cannot be directly applied to such novel WSN in CPSS. To solve this problem, we investigated the transmission optimization of the novel WSN based on CB technique in CPSS. According to the model of social dynamic mobility for social sensor nodes, we formulated the transmission optimization problem as a game and proposed a dynamic reinforcement learning algorithm based on regret matching to solve it. The corresponding implementation scheme is presented by designing the message exchange procedure. Extensive simulation results show that the proposed algorithm has better SNR performance than the LLL method and the random node selection algorithm. Moreover, we present the detailed performance analysis of the proposed algorithm for different numbers of social sensor nodes and dynamic mobility parameters. The results indicate that the proposed algorithm can better adapt to the dynamic location changes, in comparison with the existing schemes. In future work, we will consider the effects of the imperfect phase synchronization in reality. Then, we will use the approach of synchronization based on the feedback scheme and employ mobile sensors carried by people to further proceed with real-world validation of this proposed method for the CB transmission optimization in CPSS.
